# A Summary of the Contents of Exhanges

**Published:** 1888-04

**Authors:** 


### CULLINGS FROM CONTEMPORARIES.

*Edited by T. J. Bennett, M. D.*

THE "LOCO" PLANT.—Dr. Issac Ott, of Easton, Pa., writing in the *N. Y. Medical Record*, of February 18, 1888, claims to have found mydriatic properties in an aqueous solution of astragalus mollersimus—the loco plant, which abounds in West Texas. The action of this plant on horses is familiar to stock-raisers in Texas. It seems to make the animals crazy, and they leap and behave strangely. Dr. Ott believes, from recent experiments, that the narcotic action of this plant, and the dilatation of the pupil cause the leaping and other strange actions of the "locoed" animal.

A sample of the weed has been sent for, and an effort to extract its active principle will be made in the chemical laboratory of the University of Texas.

Stretching the great Sciatic.—Notwithstanding nerve stretching has been condemned as possessing no special value, Dr. D. McLean, Professor of Surgery in the University of Michigan, writing in the *American Lancet*, for February, 1888, says, he has not had a failure in ten years. Uncontrolable cases of sciatica are met with in every physician's practice. Then why not stretch the nerve? Dr. McLean cuts down upon the nerve, and stretches it with his fingers. The wound is treated antiseptically.

---

New York City contains 120 female physicians.

---

Gustave Bernutz, the distinguished gynecologist, died December 20, age 69.

---

The Telephone Bullet Probe.—February 4, Dr. J. H. Girdner, of New York, read a paper before the Academy of Medicine, in which he described his telephone bullet-probe, after one year's experience with its working. The paper appeared in the *New York Record* of above date. Great advantage is claimed for this instrument over the Nelaton probe and all others. The instrument is described as a sharp, slender steel needle, which is intended to pass through the tissues without previously preparing a tract. The vibration is not communicated by coming in contact with bone and other tissue, but instantly a sharp click is noted, when the probe touches the leaden missile. The needle may now remain with its point in contact with the ball, the handle of the instrument having been removed, and serve as a guide in cutting down on the bullet.

---

The Third Set of Triplets.—News comes from Texarkana that on the 26th of February, the wife of Mr. James McIllmore, gave birth to triplets, two boys and a girl. The couple have been married only three years, and this is the third set of triplets that has been born to them during that time, and what is unusual, they are all alive and doing well.

It is said that the McIllmore neighborhood is indifferent to the immigration movement.

---

Eighty-five Illegal Practitioners Hunted Down.—The State Medical Association of New York, has set the proper example. During the past year this organization has hunted down 85 medical humbugs, who have been imposing upon ignorant people, and getting their money. Ten were imprisoned and the other seventy-five paid fines aggregating $6,000.

A Midwife in Iowa.—The case was a breech presentation. The widwife pulled upon the scrotum until it was at least six inches long. It was presumed that consultation would have been called sooner, had the child been a female. When Dr. W. H. Carter, who related the incident, arrived upon the scene, the old lady seemed puzzled, and said "she did not see what was the matter, that she had the best of the hand-holt, but she did not know what it was."

It is said that this widwife still does a good business under the recent practice act in Iowa.

A New Hæmostatic and Uterine Tonic.—Dr. D. M. Beck, (Medical Waif, March, 1888,) calls attention to tincture of Tiger Lilly in the treatment of menorrhagia and metrorrhagia of anæmic relaxed women. The drug was first brought to the notice of the profession by Dr. I. G. M. Goss, of Georgia, in 1877.

Dr. Blanchard, of Delphi, Ind., advises that the tincture be prepared as follows:

R. Tiger Lilly Blossom, ℥ viii

Alcohol O j

Mix and macerate for two weeks. Dose, five to ten drops, three times a day.

These gentlemen recommend that its use be continued for two or three months, and prescribe it wherever ergot is indicated.

A New Operation.—The next new thing you hear of in surgery will be the total extirpation of the bladder and the transplantation of the ureters to the rectum. Novaro (*El Siglo Medico*, *Deutsche Mediz. Zeit.*, Feb. 9, 1888, *American Practitioner and News*, March 3.) has successfully performed this operation on dogs. The sphincter ani suffices to retain the urine for a time. Dogs operated on in January, 1887, are still living and are in excellent condition.

Fuchsin in Bright's Disease.—Fuchsin is the hydrochlorate of rosaniline, a dye, and is given in doses of from three to six grains, with essence of mint and syrup. The second dose is generally sufficient to reduce anasarca and the abuminuria. (*El Siglo Medio*, *Am. Pract. and News.*)

---

NEPHRORRAPHY.—Dr. G. Wilcox, of Buffalo, N. Y. (Annals of Surgery, March, 1888.) reports a successful case of nephrorraphy for fixation of a floating kidney.

The patient was a female, 24 years of age, and suffered with neuralgic and dragging pains in the kidney for nine years. When the trouble began, she had been lifting heavily, and felt something "give way in her back."

The operation consisted in making an incision midway between the last rib and the crest of the illeum, extending from the anterior edge of the quadratus lumborum forward for about four inches, cutting through the entire wall. The kidney was grasped and brought to the lips of the wound, and there secured with three heavy cat gut sutures passing through the perineval fat and each lip of the incision. The remaining portion of the wound was loosely drawn together and allowed to heal by granulation.

Antiseptic precautions were observed. The patient made a good recovery, the temperature never rising above 101 °.

The gland remained in position and there was no more pain.

This makes the twenty-third operation for fixation of floating kidney, on record. Kahn of Germany made the first success.

---

LEON BASSEREAU, author of the "Dual theory of Syphilis," died in Paris recently, age 77.

---

SUDDEN DEATH AFTER A BLOW ON THE TESTICLE.—Ivanoff records the following case: A middle-aged man was engaged in an altercation with a woman in the street, when she struck him a violent blow on the scrotum. He sank at once unconscious, and died in a few minutes, before the surgeon could arrive. At the autopsy nothing abnormal, except slight hyperæmia of the brain, was found to account for the death. Ivanoff considered death to have resulted from syncope due to the excessive pain caused by the blow on the testicles.—*N. Y. Med. Journal.*

---

THEINE IN MYALGIA.—Dr. T. J. Mays, of Philadelphia, concludes a series of observations in *Polyclinic*, for February, upon the special therapeutic indications for the use of theine. He has shown that theine, in doses from one-sixth to one-half grain, hypodermically, over the seat of the pain, relieves promptly and in nearly every instance, permanently, every character of muscular pain.

---

LEPROSY.—Many of the Medical Journals and Societies of the country are now discussing the question of "Leprosy in the United States." It is estimated by Dr. C. W. Allen, of New York, that the total number of lepers in this country is one hundred and fifty. If Texas has more than one case, the writer is not aware of it. A case was reported in Coryell connty, several years ago.

---

AMPUTATION OF THE CLITORIS.—Dr. R. St. Clair, of Brooklyn, removed by galvanic cautery knife, an elongated and sensative clitoris from a young lady, 21 years of age, who had suffered with states of orgasm, occasioned by the least amount of exercise either walking or riding, since she was 18 years of age. The result of the operation was perfect recovery. The patient has since married and borne two or three children, but has but little if any passion. The case is reported in the January number of the *Med. Summary*.

---

AN ADVANCE IN SURGICAL DRESSINGS.—Five parts of either Tartaric or Hydrochloric Acid added to a 1 to 1,000 solution of the Bichloride of Mercury, will prevent the solution from forming a precipitate when it comes in contact with an albuminous fluid.—*E. Laplace, in N. O. Medical and Surgical Jonrnal.*

---

ALBUMINURIA IN THE INSANE.—According to recent investigations 40 per cent. of insane and epileptic patients have albuminuria.

---

DIFFERENTIATION BETWEEN RHEUMATISM AND GOUT.—A Frenchman who was troubled with gout was asked what difference there was between that complaint and rheumatism. "One very great difference," replied Monsieur. "Suppose you take one vise, put your finger in; you turn de screw till you can bear him no longer—dat is de rheumatism; den s'pose you give him one turn more—dat is de gout!"

---

CHILDBIRTH IN A RAILWAY CAR.—A woman who expected to be confined in a week or ten days, was riding on a car which was running at the rate of twenty miles an hour. She felt an inclination to go to the water-closet, when a few pains came on, and she heard something drop. Her groans attracted the obliging conductor, who stopped the train, went back and found the child, which was alive, though somewhat bruised about the head. It had dropped through the hole in the closet.—*Dr. Wm. Osler, in the Canada Medical and Surgical Journal, January, 1888.*

---

PASTEUR.—The Emperor of Austria is about to confer upon Pasteur the Order of Iron Crown, a distinction which carries with it the right to the title of Baron.—*New England Medical Monthly, February 15.*

---

CINDER IN THE EYE.—It is advised by those who have tried it hundreds of times, that rubbing the well eye will remove the cinder from the other eye in two minutes. Try it.

