# A Correction

**Published:** 1888-06

**Authors:** 


A Correction.—Under the head of *Nephrorrhaphy*, in the April number of this Journal, appeared an extract from the *Annals of Surgery* for March in which credit was given E. Kalm, of Germany, for the first operation for fixation of the floating kidney. The article, from which the extract referred to was taken, was written by Dr. Dewitt G. Wilcox, of Buffalo, N. Y. Credit should have been given to Dr. Greenville Dowell. of Galveston, Texas, now deceased. He performed the operation a number of times in 1879. Kahm did not undertake the operation until 1881. See Reference Handbook, 4 vol., 301.

